# Mitochondrial DNA Leakage Caused by *Streptococcus pneumoniae* Hydrogen Peroxide Promotes Type I IFN Expression in Lung Cells

**DOI:** 10.3389/fmicb.2019.00630

**Published:** 2019-03-28

**Authors:** Yue Gao, Wenchun Xu, Xiaoyun Dou, Hong Wang, Xuemei Zhang, Shenghui Yang, Hongyi Liao, Xuexue Hu, Hong Wang

**Affiliations:** ^1^Key Laboratory of Diagnostic Medicine Designated by the Ministry of Education, Chongqing Medical University, Chongqing, China; ^2^School of Laboratory Medicine, Chongqing Medical University, Chongqing, China; ^3^Institute of Life Sciences, Chongqing Medical University, Chongqing, China

**Keywords:** *Streptococcus pneumoniae*, hydrogen peroxide, mitochondrial damage, mtDNA, IFNβ, STING

## Abstract

*Streptococcus pneumoniae (S. pn)*, the bacterial pathogen responsible for invasive pneumococcal diseases, is capable of producing substantial amounts of hydrogen peroxide. However, the impact of *S. pn*-secreted hydrogen peroxide (H_2_O_2_) on the host immune processes is not completely understood. Here, we demonstrated that *S. pn*-secreted H_2_O_2_ caused mitochondrial damage and severe histopathological damage in mouse lung tissue. Additionally, *S. pn*-secreted H_2_O_2_ caused not only oxidative damage to mitochondrial deoxyribonucleic acid (mtDNA), but also a reduction in the mtDNA content in alveolar epithelia cells. This resulted in the release of mtDNA into the cytoplasm, which subsequently induced type I interferons (IFN-I) expression. We also determined that stimulator of interferon genes (STING) signaling was probably involved in *S. pn* H_2_O_2_-inducing IFN-I expression in response to mtDNA damaged by *S. pn*-secreted H_2_O_2_. In conclusion, our study demonstrated that H_2_O_2_ produced by *S. pn* resulted in mtDNA leakage from damaged mitochondria and IFN-I production in alveolar epithelia cells, and STING may be required in this process, and this is a novel mitochondrial damage mechanism by which *S. pn* potentiates the IFN-I cascade in *S. pn* infection.

## Introduction

*Streptococcus pneumoniae (S. pn)*, a member of the human nasopharyngeal microbiota, can cause pneumonia and other invasive pneumococcal diseases, such as otitis media, meningitis and bacteremia, especially in children and the elderly (Weiser et al., 2018). The virulence of *S. pn* is known to be dependent upon several factors, including its polysaccharide capsule, surface proteins, enzymes, and the cholesterol-dependent cytolysin, pneumolysin (Ply) (Mitchell and Mitchell, 2010). These virulence factors play an important role in the invasion of *S. pn* into the host. Interestingly, *S. pn* can secrete a substantial amount of hydrogen peroxide (H_2_O_2_) via an enzymatic reaction catalyzed by pyruvate oxidase, SpxB, during the aerobic metabolism. The *spxB* gene is not only a virulence determinant in *S. pn*, but it is also essential for resistance to the toxic by-product produced by itself (Li-Korotky et al., 2009). It can influence the synthesis of acetyl-phosphate, a potential source of ATP under the aerobic conditions in *S. pn* (Pericone et al., 2003). Meanwhile, it can affect the sugar utilization pattern and capsule biosynthesis (Carvalho et al., 2013). Research has shown that strains of *S. pn* that lack *spxB* during growth produce significantly reduced levels of H_2_O_2_ (Yesilkaya et al., 2013; Echlin et al., 2016). And compared with the wide type *S. pn*, the *spxB* mutant strain showed reduced virulence in animal models for nasopharyngeal colonization and pneumonia (Spellerberg et al., 1996). Through its ability to produce H_2_O_2_, *S. pn* is able to not only induce autolysis (Regev-Yochay et al., 2007), but also inhibit a variety of competing organisms, such as *Haemophilus influenzae* (Pericone et al., 2000) and *Staphylococcus aureus* (Regev-Yochay et al., 2006) in the aerobic environment of the respiratory tract.

Previous research has shown that *S. pn*-secreted H_2_O_2_ influences the host physiology and immune defense. During pneumococcal meningitis, both *S. pn*-secreted H_2_O_2_ and Ply are sufficient to induce mitochondrial damage, trigger the release of apoptosis-inducing factor (AIF) from mitochondria, and ultimately mediate apoptosis (Braun et al., 2002). *S. pn*-secreted H_2_O_2_ has been shown to induce endoplasmic reticulum (ER) stress, activate the mitogen-associated protein kinase (MAPK) signaling pathways, and regulate target genes (Loose et al., 2015). Furthermore, H_2_O_2_ secreted by *S. pn* is required for the induction of cardiomyocyte cell death, which is involved in the pathogenesis of *S. pn* infection in the heart (Brissac et al., 2017). However, the mechanism of how *S. pn*-secreted H_2_O_2_ activates the immune system are not fully understood in acute pneumonia.

Mitochondrial deoxyribonucleic acid (mtDNA) is an important damage-associated molecular pattern (DAMP), which contains a large number of unmethylated CpG sequences (Nakayama and Otsu, 2018). It is thought that mtDNA is more susceptible to damage, owing to an inefficient DNA repair mechanism and the lack of protective histone packaging. Damaged mtDNA released to into the cytoplasm or circulation has been shown to induce the transcription of pro-inflammatory cytokines, including MMP-8, TNFα, IL-6, and IL-1β (Fang et al., 2016). Moreover, mtDNA is involved in the induction of endothelial inflammation (Mao et al., 2017) and cardiomyocyte ischemia/reperfusion-injury (Hu et al., 2018). Previous research has confirmed that *S. pn*-secreted H_2_O_2_ leads to cytotoxic DNA damage in lung cells (Rai et al., 2015). However, the source of this oxidative DNA damage and whether it is involved in the host immune response remains unclear.

The ability to sense aberrant nucleic acids is a cornerstone of the innate immune system against pathogens. Stimulator of interferon genes (STING), a key innate immune signaling adaptor, responds to various forms of DNA species, including self-DNA from the nucleus of damaged cells. Self-DNA may cause various autoimmune diseases such as systemic lupus erythematosus (SLE) (Barber, 2015). Certain bacteria, such as *Listeria monocytogenes*, secrete cyclic dinucleotides (CDNs) that induce STING signaling within the host (Sauer et al., 2011). Similarly, *S. pn* DNA stimulates type I interferons (IFN-I) (IFNα, IFNβ) production in a STING-dependent manner (Parker et al., 2011; Koppe et al., 2012). In addition, mtDNA, which exists as a closed circular doubles-stranded DNA species, is able to activate STING, resulting in the upregulation of IFN-I and other interferon-stimulated genes (ISGs), under cellular damage and stress (Fang et al., 2016). However, there is little data to indicate mtDNA damaged by *S. pn*-secreted H_2_O_2_ induces IFN-I expression.

In this study, we demonstrated that *S. pn*-secreted H_2_O_2_ is capable of causing mitochondrial damage and mtDNA leakage into the cytosol of human alveolar epithelial cells, which further trigger the expression of IFNβ mediated by STING signaling probably. Thus, this study revealed a new strategy by which *S. pn* activates the host immune response.

## Materials and Methods

### Ethics Statement

All animal experiments in this study were conducted in accordance with the guidelines of the Institutional Animal Care and Use Committee of Chongqing Medical University and were authorized by the Animal Ethics Committee of Chongqing Medical University.

### Bacterial Strains and Culture Conditions

The *S. pn* standard strain D39 (NCTC 7466, serotype 2) was purchased from the National Collection of Type Cultures (London, United Kingdom). The *S. pn* spxB knockout mutant strain (D39ΔspxB) was constructed by long flanking homology-polymerase chain reaction (LFH-PCR), as described before (Wu et al., 2014). Briefly, the *spxB* gene was substituted with an erythromycin resistant sequence. The positive clones were selected on blood agar plates containing 0.25 μg/ml erythromycin and identified by PCR. All *S. pn* strains were grown in C plus Y medium at 37°C in 5% CO_2_ until the optical density at 600 nm equaled 0.5 (OD600 = 0.5).

### Mouse Model of Acute Pneumonia

Female C57BL/6 mice (6–8 weeks old) were purchased from Chongqing Medical University (Chongqing, China) and were maintained under specific-pathogen-free conditions in a temperature-controlled room of the animal facility at Chongqing Medical University. All animal experiments were approved by the respective ethics committees of Chongqing Medical University. Mice were inoculated intranasally with 1 × 10^8^ CFU of D39 (NCTC 7466, serotype 2) or D39Δ*spxB* in 30 μL of sterile PBS (*n* = 5 mice/group). Catalase was given intravenously (at 6, 12, 18, 22, 23, and 24 h) in the other five mice inoculated with 1 × 10^8^ CFU of D39. Mice were sacrificed 24 h post-infection, and blood and lung homogenate supernatants were collected.

### Cell Culture

The human lung alveolar carcinoma (type II pneumocyte) A549 cell lines were cultured in DMEM (Hyclone, United States) supplemented with 10% fetal bovine serum (FBS) (BI, United States) and 1% penicillin-streptomycin (Hyclone, United States) at 37°C in 5% CO_2_. Wild-type (WT) and STING knockout mouse embryonic fibroblast (MEF sting−/−) cells were also cultured in DMEM supplemented with 10% FBS (Gibco, United States) and 1% penicillin-streptomycin at 37°C with 5% CO_2_. MEF sting−/− cells were kindly provided by Professor Chen Wang (School of Life Sciences and Technology, China Pharmaceutical University, Nanjing, China) (Lu et al., 2018). In order to construct a mtDNA-depleted cell line, A549 cells were cultured in DMEM supplemented with 10% FBS, 1% penicillin-streptomycin, and ethidium bromide (EtBr) (300 ng/ml) for 5 days at 37°C in 5% CO_2_.

### Mitochondrial Transmembrane Potential (ΔΨm) Assay

Mitochondrial transmembrane potential was assessed using a JC-1 kit (Solarbio, China). JC-1 is a fluorescent probe that indicates mitochondrial membrane potential loss. In normal cells, JC-1 aggregates in intact mitochondria (red fluorescence) but becomes a monomer (green fluorescence) in cells with disrupted mitochondrial membrane. After incubation with specific stimulators, including D39 with or without catalase (Cat) and D39Δ*spxB* (MOI = 200) at 2 h. A549 cells were incubated in DMEM containing 10 μM JC-1 at 37°C and protected from light for 20 min. The cells were then washed with ice-cold 1× JC-1 staining buffer twice and then imaged with a fluorescent microscope (Nikon ECLIPSE 80i, Japan).

### Lung Histology and Immunohistochemistry

Lung tissue was removed and fixed in 4% paraformaldehyde. The tissues were then embedded in paraffin, and then 5-μm sections were cut. The sections were stained with hematoxylin and eosin (Sigma-Aldrich, United States) and analyzed using a light microscope (Nikon ECLIPSE 80i, Japan). The degree of peribronchial inflammation was semi-quantitatively graded as described methods (Blanquiceth et al., 2016). The tissues were scored as follows: 0, normal; 1, a few cells; 2, a ring of cells 1 cell layer deep; 3, a ring of cells 2–4 cells deep; 4, a ring of cells 5–6 cells deep; and 5, a ring of cells of > 6 cells deep.

For immunohistochemistry of lung tissue sections, citrate buffer was used for antigen retrieval. Lung sections were then incubated with an anti-PINK1 antibody (Novus Biologicals, United States), according to standard protocols. The mean integrated optical density (IODs) of PINK1 expression was measured using Image-Pro Plus (Media Cybernetics, Silver Spring, MD, United States).

### Transmission Electron Microscopy

A549 cells were infected with D39 or D39Δ*spxB* for 2 h. The cells were then harvested, washed with sterile PBS twice, and fixed with ice-cold 4% glutaraldehyde. Fixed cells were sectioned according to the Electron Microscopy Research Service of Chongqing Medical University and observed with a Hitachi H-7500 transmission electron microscope (Hitachi, Japan).

### Extraction of Cytoplasmic DNA and Transfection

Cytoplasmic DNA was extracted as previously described (Holden and Horton, 2009). Briefly, 2 × 10^6^ cells were stimulated as indicated. The cells were then harvested, washed with phosphate-buffered saline (PBS), and then mixed on a rotator in 500 μl of digitonin solution (25 μg/ml) containing 150 mM NaCl and 50 mM HEPES (pH 7.4) for 30 min at room temperature. The lysate was then centrifuged at 1000 × *g* for 5 min thrice in order to remove the nuclei and intact cells. The supernatant was transferred to a new tube and then centrifuged at 17,000 × *g* for 10 min in order to pellet the remaining cellular debris. Total cytosolic DNA were extracted using a DNA Blood Mini Kit (Qiagen, Germany), precipitated with 100 μl absolute ethanol, and stored at −20°C. A549 cells were transfected with the cytosolic DNA for 6 h using Lipofectamine 2000 (Lipo 2000) (Invitrogen, United States) according to the manufacturer’s instructions.

### mtDNA Copy Number and Transcription Level

In order to quantify mtDNA copy number, total DNA was extracted from the lungs of mice and A549 cells using a DNeasy Blood & Tissue Kit (Qiagen, Germany). For the quantification of mtDNA transcription level, total RNA was extracted from the lungs of mice and A549 cells using RNAiso plus reagent (Takara Bio, China) following the manufacturer’s instructions. Cytochrome B (*Cytb*) and cytochrome c oxidase subunit 3 (*CoxIII*) were amplified in order to analyze the copy number and transcription level of mtDNA. G*apdh* was used as the internal control (Hu et al., 2018). The primers used in this study are listed in Table 1.

**Table 1:** The sequences of PCR primers.

Gene	Orientation	Sequence
Human-*GAPDH*	Sense	5′-GAAGGGCTCATGACCACAGT-3′
	Anti-sense	5′-GGATGCAGGGATGATGTTCT-3′
Human-*IFNβ*	Sense	5′-AGATCAACCTCACCTCAGG-3′
	Anti-sense	5′-TCAGAAACACTGTCTGCTGG-3′
Human-*IFNa2*	Sense	5′-CCTGATGAAGGAGGACTCCATT-3′
	Anti-sense	5′-AAAAAGGTGAGCTGGCATACG-3
Human-*IFNa5*	Sense	5′-GTACTAGTCAATGAGAATCATTTCG-3′
	Anti-sense	5′-TCCTCTGATGAATGTGGACTCT-3′
Human-*COXIII*	Sense	5′-CTCTGGACCCTACCGACTT-3′
	Anti-sense	5′-CAGCCAGGGCAGTAA-3′
Human-*ISG15*	Sense	5′-GAGAGGCAGCGAACTCATCT-3′
	Anti-sense	5′-CTTCAGCTCTGACACCGACA-3′
Human-*OASL-1*	Sense	5′-CCATCACGGTCACCATTGTG-3′
	Anti-sense	5′-ACCGCAGGCCTTGATCAG-3′
Human-*RNF185*	Sense	5′-AGGACCCCAGAGAGAAGACC-3′
	Anti-sense	5′-CAATTCCAAAAGACATCTGG-3′
Mouse-*Gapdh*	Sense	5′-CGGAGTCAACGGATTTGGTC-3′
	Anti-sense	5′-GACAAGCTTCCCGTTCTCAG-3′
Mouse-*Ifnβ*	Sense	5′-ATTGCCTCAAGGACAGGATG-3′
	Anti-sense	5′-GGCCTTCAGGTAATGCAGAA-3′
Mouse-*Pgc1-α*	Sense	5′-TATGGAGTGACATAGAGTGTGCT-3′
	Anti-sense	5′-CCACTTCAATCCACCCAGAAAG-3′
Mouse-*Cxcl10*	Sense	5′-CCTGCCCACGTGTTGAGAT-3′
	Anti-sense	5′-TGATGGTCTTAGATTCCGGATTC-3′
Mouse-*Sting*	Sense	5′-GAGAGCCACCAGAGCACAC-3′
	Anti-sense	5′-CGCACAGTCCTCCAGTAGC-3′
Mouse*-Cytb*	Sense	5′-CTCACAGGACTGGCGAGAC-3′
	Anti-sense	5′-ACAGCCCCAATGACCCTCA-3′
Mouse*-CoxIII*	Sense	5′-TGCTGACCTCCAACAGGAAT-3′
	Anti-sense	5′-GTCCATGGAATCCAGTAGCCA-3′

### Immunofluorescence and Confocal Imaging

Treated A549 cells were incubated with MitoTracker^®^ Red CMXRos (Yeasen, China) (mitochondrial red fluorescent probe) at 37°C for 45 min. Cells were fixed, permeabilized with 0.1% Triton X-100, blocked with 10% donkey serum, and then incubated with an anti-8-hydroxyguanine (8-OHdG) antibody (Santa Cruz Biotechnology, United States) at 4°C overnight. Then the cells were incubated with fluorescent-labeled secondary antibodies (Bioss, China). Nuclei were stained with DAPI (Beyotime, China) at room temperature for 15 min. Cells were observed with a Nikon ECLIPSE Ti confocal microscope (Nikon, Japan). The images were captured and analyzed by using NIS-Elements Viewer (Nikon, Japan).

### Western Blot Analysis

A549 cells were lysed with RIPA buffer (Beyotime, China) supplemented with PMSF and phosphatase inhibitor (100:1:1) (BioTools, United States). Protein samples were separated onto sodium dodecyl sulfate (SDS)-polyacrylamide gels and then transferred onto PVDF membranes (Millipore, United States). The membranes were incubated with a primary antibody overnight at 4°C, including anti-human STING and anti-human GAPDH (Cell Signaling Technology, United States). The membranes were then incubated with an HRP-conjugated secondary antibody for 1 h at 37°C. Bands were visualized by using Image Lab (Bio-Rad Laboratories, Hercules, CA, United States). GAPDH was used as a loading control.

### Real-Time PCR

Total RNA was extracted from the lungs of mice and cells using RNAiso plus reagent (Takara Bio, China), according to the manufacturer’s protocol. The mRNA was then reverse transcribed into cDNA using the PrimeScript^TM^ RT reagent kit (Takara Bio, China). All real-time PCR reactions were performed using TB Green Premix Ex TaqTM II on a Bio-Rad CFX-96 cycler (Bio-Rad Laboratories, United States). The expression of mRNA was normalized against GAPDH. The data shown are representative of three separate experiments. The primers used in this study are listed in Table 1.

### ELISA

Cytokine levels in the blood, lung homogenate supernatants, and cell culture supernatants were measured by using ELISA assays, according to the manufacturer’s instructions. The following ELISA kits were used in this study: LEGEND MAX^TM^ Mouse IFNβ (Biolegend, United States) and Human IFNβ (Cloud-clone, China).

### Statistical Analysis

All experiments were performed at least three times, and the data are presented as the mean ± SD. Student’s *t*-test was used for statistical analysis, and GraphPad Prism 5 software was used to perform statistical analysis for all experiments. *P* < 0.05 was considered statistically significant.

## Results

### H_2_O_2_ Secreted by *S. pn* Induces IFN-I Expression *in vivo* and *in vitro*

**FIGURE 1:**
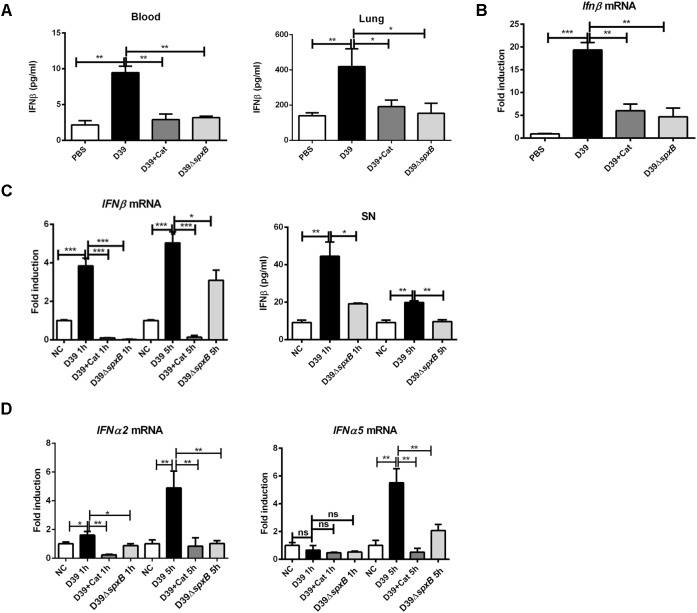
*S. pn*-secreted H_2_O_2_ could augment the expression of IFN-I *in vivo and in vitro*. Female C57BL/6 mice were intranasally infected with D39 and D39Δ*spxB* (1 × 10^8^ CFU) for 24 h, catalase was given intravenously (at 6, 12, 18, 22, 23, and 24 h) in the other five mice inoculated with 1 × 10^8^ CFU of D39. **(A)** The production of IFNβ in blood and lung homogenates were measured by ELISA. **(B)** The expression of *Ifnβ* in lungs were analyzed by real-time PCR. **(C,D)** A549 cells were infected with D39 with or without catalase (Cat) and D39Δ*spxB* (MOI = 200) at 1 and 5 h, *IFNβ*, (**C**, left panel) *IFNa2* and *IFNa5* **(D)** mRNA levels were determined by real-time PCR. **(C)** A549 cells were infected with D39 and D39Δ*spxB* (MOI = 200) at 1 and 5 h, IFNβ in supernatants (SN) was quantified by ELISA (right panel). NC, negative control. All data were presented as means ± SD from three independent experiments. ^∗^*P* < 0.05; ^∗∗^*P* < 0.01; ^∗∗∗^*P* < 0.001 was considered statistically significant and highly statistically significant differences, respectively; ns, not significant.

In order to assess whether H_2_O_2_ secreted by *S. pn* induces the production of IFN-I *in vivo*, we used a mouse model of acute pneumonia. C57BL/6 mice were intranasally infected with *S. pn* D39 or D39Δ*spxB* for 24 h, catalase was given intravenously in the other five mice inoculated with 1 × 10^8^ CFU of D39. ELISA assays demonstrated significant upregulation of IFNβ in the blood and lung homogenate supernatants of D39-infected mice compared to D39Δ*spxB*-infected mice and catalase treatment of D39-infected mice (Figure 1A). Similarly, *Ifnβ* mRNA levels were increased in the lung tissue of D39-infected mice, but not in the lung tissues of D39Δ*spxB*-infected mice and catalase treatment of D39-infected mice (Figure 1B). We also infected the human alveolar epithelial cell line A549 with D39 or D39Δ*spxB* and analyzed the expression of IFN-I at 1 and 5 h post-infection. D39 infection was capable of inducing expression of *IFNβ* and *IFNa2* (subtype of IFN-I) at both 1 and 5 h compared to D39Δ*spxB* infection. Pre-treatment of A549 cells with catalase prior to D39 infection resulted in diminished *IFNβ* and *IFNa2* transcript levels, in which 5-fold reduction was observed in *IFNβ* and *IFNa2* transcript levels at 5 h post-infection (Figure 1C,D, left panel). We also found that the production of IFNβ was reduced by 50% in the supernatant of A549 cells infected with D39Δ*spxB* as compared to D39-infected cells (Figure 1C, right panel). We further determined that D39 infection increased the expression of *IFNa5* (subtype of IFN-I) at 5 h, but not 1 h post-infection (Figure 1D, right panel).

Together, these data suggest that H_2_O_2_ secreted by *S. pn* is capable of inducing IFN-I expression in lung cells.

### *S. pn*-Secreted H_2_O_2_ Is Sufficient to Induce Mitochondrial Dysfunction

**FIGURE 2:**
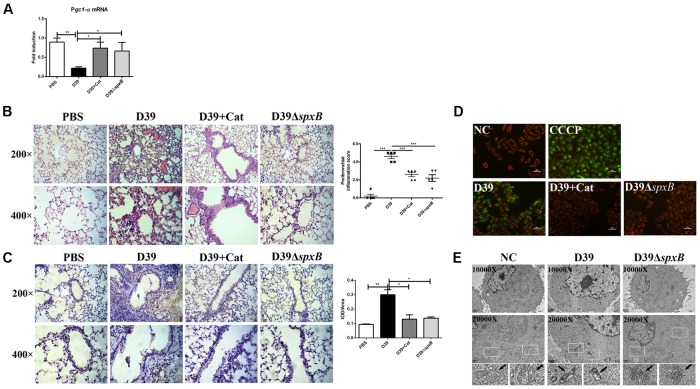
*S. pn*-secreted H_2_O_2_ led to the mitochondrial malfunction in lung cells. Female C57BL/6 mice were intranasally infected with D39 and D39Δ*spxB* (1 × 10^8^ CFU) for 24 h, catalase was given intravenously (at 6, 12, 18, 22, 23, and 24 h) in the other five mice inoculated with 1 × 10^8^ CFU of D39. **(A)** The expression of *Pgc1-α* in lungs were analyzed by real-time PCR. **(B)** The expression of PINK1 in lung section of mouse were analyzed by immunohistochemistry (left panel). Score of PINK1 production was measured using the scale described in Section “Materials and Methods” (right panel). **(C)** Pathological analyses were done by hematoxylin and eosin staining, with lung sections examined under light microscopy at 200× (scale bar = 100 μm) and 400× (scale bar = 50 μm) magnification (left panel). Score of peribronchial inflammation was measured using the scale described in Section “Materials and Methods” (right panel). **(D)** A549 cells were infected with D39 with or without catalase (Cat) and D39Δ*spxB* (MOI = 200) for 2 h, ΔΨm was measured using JC-1 probe. CCCP were applied as a positive control. Scale bar = 50 μm. **(E)** Changes of ultrastructure of A549 cells exposed to D39 and D39Δ*spxB* (MOI = 200) for 2 h were monitored by transmission electron microscopy. NC, negative control. All data were presented as means ± SD from three independent experiments. ^∗^*P* < 0.05; ^∗∗^*P* < 0.01; ^∗∗∗^*P* < 0.001 was considered statistically significant and highly statistically significant differences, respectively; ns, not significant.

Given that IFNβ production is triggered by mtDNA following mitochondrial stress (Fang et al., 2016), we theorized that H_2_O_2_ generated by *S. pn* induces mitochondrial damage in mouse lung tissue. The mRNA level of peroxisome proliferator-activated receptor γ (PPARγ) coactivator 1α (*Pgc1-α*), which is the master regulator of mitochondrial biogenesis and functions as a transcriptional co-regulator (Dorn et al., 2015), was markedly decreased by 75% in D39-infected mice, but not in D39Δ*spxB*-infected mice and catalase treatment of D39-infected mice (Figure 2A). PTEN-induced putative kinase 1 (PINK1) is a regulator of mitophagy, which is rapidly degraded when mitochondria are healthy, but accumulates on the surface of damaged mitochondria (Jin et al., 2010). We observed by immunohistochemistry that infection with D39 significantly induced the accumulation of PINK1 on the mitochondria in the lung tissue of mice, while both infection with D39Δ*spxB* and catalase treatment of D39 infection did not increase PINK1-mitochondria interactions (Figure 2B). In addition, morphological and histopathological analyses of murine lung tissue revealed that severe pulmonary injuries were found in the D39-infected group, with obvious hemorrhage and massive inflammatory cell infiltration in the peribronchial, as compared to the D39Δ*spxB*-infected group and these pulmonary injuries were not markedly observed in the lung tissue of mice infected with D39 pre-treatment with catalase (Figure 2C). These data suggest that *S. pn*-secreted H_2_O_2_ causes mitochondrial damage in the lungs of mice.

To further understand the extent of mitochondrial damage induced by the H_2_O_2_ generated by *S. pn*, we assessed mitochondrial function in A549 cells infected with *S. pn* by measuring the ΔΨm. We used an oxidative phosphorylation uncoupler, carbonyl cyanide-*m*-chlorophenylhydrazone (CCCP), as a positive control, which causes depolarization of mitochondria and mitochondrial damage (Park et al., 2018). Our results showed an increase in green fluorescence in A549 cells after infection with D39 for 2 h, indicating that the ΔΨm values significantly decreased. Moreover, the addition of catalase significantly attenuated the decrease in the ΔΨm values induced by D39 infection (Figure 2D). Similarly, we used transmission electron microscopy to monitor changes in mitochondria morphology. We observed abnormal mitochondrial morphology in A549 cells infected with D39, including mitochondrial swelling, condensation, and abnormal cristae shape. However, these changes were not observed in D39Δ*spxB*-infected cells (Figure 2E). These results suggest that *S. pn*-secreted H_2_O_2_ induces mitochondrial damage in alveolar epithelial cells.

Taken together, our results demonstrate that H_2_O_2_ produced by *S. pn* causes mitochondrial dysfunction in lung cells both *in vivo* and *in vitro*.

### *S. pn*-Secreted H_2_O_2_ Mediates Oxidative Damage of Mitochondrial DNA

**FIGURE 3:**
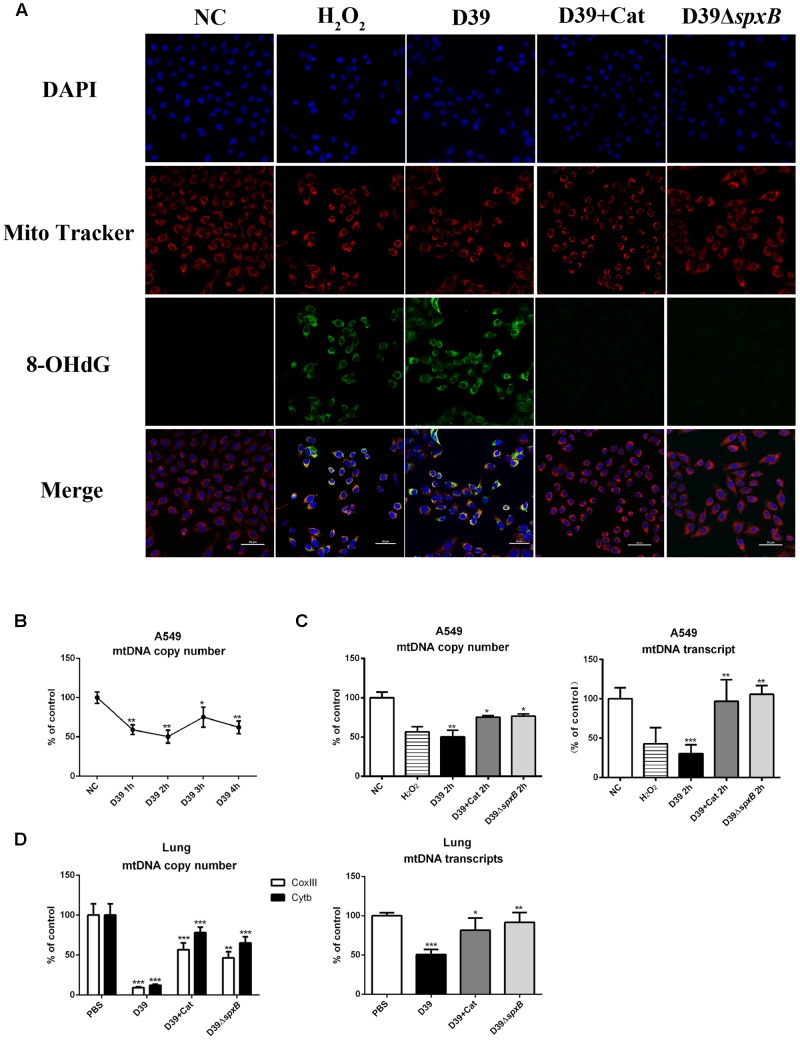
*S. pn*-secreted H_2_O_2_ caused oxidative damage of mitochondrial DNA. **(A)** A549 cells were stimulated with D39 with or without catalase (Cat) and D39Δ*spxB* (MOI = 200), as well as 1 mM H_2_O_2_ for 2 h, representative images of MitoTracker red (red), 8-OHdG immunostaining (green), and DAPI (blue) in A549 cells. The orange in the merged images of green and red fluorescence indicates 8-OHdG-positive cells. **(B)** A549 cells were infected with D39 (MOI = 200) at indicated time points, mtDNA copy number was analyzed by real-time PCR. **(C)** A549 cells were stimulated with D39 with or without catalase (Cat) and D39Δ*spxB* (MOI = 200), as well as 1 mM H_2_O_2_ for 2 h, mtDNA copy number (left panel) and mtDNA transcript level (right panel) was analyzed by real-time PCR. **(D)** Female C57BL/6 mice were intranasally infected with D39 and D39Δ*spxB* (1 × 10^8^ CFU) for 24 h, catalase was given intravenously (at 6, 12, 18, 22, 23, and 24 h) in the other five mice inoculated with 1 × 10^8^ CFU of D39. mtDNA copy number (left panel) and mtDNA transcript level (right panel) were analyzed by real-time PCR. mtDNA level was normalized to the internal control GAPDH. Mitochondrial genes *CoxIII* and *Cytb* were chosen to indicate mtDNA transcription. NC, negative control. All data were presented as means ± SD from three independent experiments. ^∗^*P* < 0.05; ^∗∗^*P* < 0.01; ^∗∗∗^*P* < 0.001 was considered statistically significant and highly statistically significant differences, respectively; ns, not significant.

The expression of 8-hydroxyguanine (8-OHdG) is known to be reflective of oxidative DNA damage. To further clarify if mtDNA is damaged by *S. pn*-secreted H_2_O_2_, we evaluated the level of 8-OHdG in A549 cells infected with D39 by immunofluorescence analysis. The number of 8-OHdG-positive A549 cells significantly increased following D39 infection, but not D39Δ*spxB* infection (Figure 3A). Furthermore, the addition of catalase markedly reduced the number of 8-OHdG-positive A549 cells infected with D39. These results suggest that *S. pn*-secreted H_2_O_2_ causes oxidative damage to the mtDNA in A549 cells, and catalase pre-treatment may prevent this phenomenon. We also explored the mtDNA copy number in A549 cells infected with D39 at 2 h post-infection. Real-time PCR analysis demonstrated that the mtDNA copy number significantly reduced by 50% over time in A549 cells after D39 infection (Figure 3B). Furthermore, the reduction in mtDNA copy number and mtDNA transcript level in D39-infected A549 cells was largely prevented by catalase pre-treatment, which is consistent with our data showing that catalase pre-treatment also decreased 8-OHdG levels in mtDNA after D39 infection. In D39Δ*spxB*-infected cells, the mtDNA transcript level and copy number were partially restored as compared with D39 infection. As expected, when A549 cells were exposed to 1 mM H_2_O_2_, we observed a 40% reduction in mtDNA copy number and a 60% reduction mtDNA transcript level by real-time PCR (Figure 3C). Likewise, copy number and transcript level of mtDNA were significantly decreased in D39-infected mouse lung tissue as compared with D39Δ*spxB*-infected mice and catalase treatment of D39 infected mice (Figure 3D).

In short, these results provide evidence that H_2_O_2_ secreted by *S. pn* induces significant oxidative damage in the mtDNA of lung cells.

### *S. pn*-Secreted H_2_O_2_ Promotes Mitochondria DNA Leakage and the Induction of IFN-I

**FIGURE 4:**
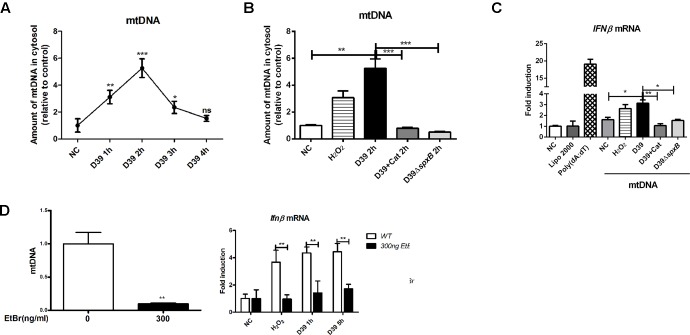
mtDNA leakage caused by *S. pn*-secreted H_2_O_2_ was involved in IFN-I induction. **(A)** A549 cells were infected with D39 (MOI = 200) at indicated time points, DNA in the cytosolic fraction was isolated, and the copy number of mtDNA (mtDNA sequences as primers) was measured and normalized with the copy number of nuclear DNA (nuclear DNA sequences as primers). **(B)** A549 cells were stimulated with D39 with or without catalase (Cat) and D39Δ*spxB* (MOI = 200), as well as 1 mM H_2_O_2_ for 2 h, DNA in the cytosolic fraction was isolated, and the copy number of mtDNA was measured and normalized with GAPDH. **(C)** A549 cells were transfected with cytosolic DNA isolated from different stimulations, including D39 with or without catalase (Cat) and D39Δ*spxB*, as well as H_2_O_2_, *IFNβ* mRNA level were determined by real-time PCR. POLY (dA: dT) (2 μg/ml) was applied as positive control. **(D)** mtDNA in A549 cells were evaluated by real-time PCR after being treated with EtBr (300 ng/ml) for 5 days (left panel). *IFNβ* mRNA level in A549 cells treated with 1 mM H_2_O_2_ or D39 (MOI = 200) were determined by real-time PCR (right panel). NC, negative control. All data were presented as means ± SD from three independent experiments. ^∗^*P* < 0.05; ^∗∗^*P* < 0.01; ^∗∗∗^*P* < 0.001 was considered statistically significant and highly statistically significant differences, respectively; ns, not significant.

To address whether *S. pn*-secreted H_2_O_2_ could lead to the leakage of mtDNA into the cytoplasm, we assessed the cytosolic mtDNA levels in D39-infected A549 cells. We show that D39 infection significantly elevated the level of mtDNA in the cytoplasm within the first 3 h post-infection. A549 cells infected with D39Δ*spxB* did not exhibit this increase in mtDNA levels in the cytoplasm (Figure 4A). As expected, catalase pre-treatment inhibited D39-induced mtDNA leakage into the cytoplasm (Figure 4B). These findings suggest that H_2_O_2_ generated by *S. pn* triggers mtDNA leakage into the cytoplasm of A549 cells.

We next sought to determine if IFN-I expression is induced by mtDNA damage caused specifically by *S. pn*-secreted H_2_O_2_ to mtDNA. Thus, we isolated mtDNA from the cytoplasm of A549 cells subjected to various stimulations, including D39 with or without catalase, D39Δ*spxB* and H_2_O_2_. We then exposed untreated A549 cells with the isolated mtDNA. Poly (dA:dT), a synthetic double-stranded DNA sequence, was used as a positive control. The cytosolic mtDNA isolated from D39-infected cells caused an upregulation in the transcription level of *IFNβ*, and a similar result was obtained with mtDNA from H_2_O_2_-stimulated cells. However, there was no increase in the expression of *IFNβ* in A549 cells treated with mtDNA from D39-infected A549 cells pretreated with catalase or D39Δ*spxB*-infected A549 cells (Figure 4C).

In order to ascertain the importance of mitochondria in the induction of *IFNβ* expression in D39-infected A549 cells, we constructed mtDNA-depleted A549 cells. A549 cells were exposed to low concentrations of ethidium bromide over time to effectively reduce the mtDNA content within these cells. Real-time PCR results confirmed the successful construction of mtDNA-deficient cells (Figure 4D, left panel). The expression of *IFNβ* in mtDNA-deficient cells was reduced by about 60% than that in WT cells following treatment with D39 or H_2_O_2_ (Figure 4D, right panel), which suggests that mtDNA plays a critical role in *S. pn* H_2_O_2_-induced production of IFNβ.

### STING Signaling Is Probably Involved in the Activation of IFN-I by S. *pn*-Secreted H_2_O_2_

**FIGURE 5:**
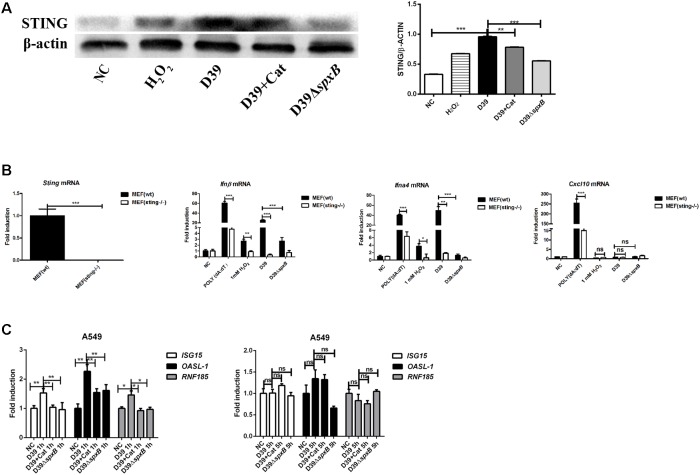
STING signaling probably participated in IFN-I activation induced by *S. pn*-secreted H_2_O_2_. **(A)** Western blot analysis of STING in A549 cells stimulated with D39 with or without catalase (Cat) and D39Δ*spxB* (MOI = 200), as well as 1 mM H_2_O_2_ for 1 h. **(B)** STING in MEF (wt) and MEF (sting–/–) were determined by real-time PCR. Transcription level of *Ifnβ*, *Ifna4* and *Cxcl10* in MEF (wt) and MEF (sting–/–) were measured by real-time PCR after being treated with D39, D39Δ*spxB* (MOI = 200) and 1 mM H_2_O_2_ for 1 h. POLY (dA: dT) (2 μg/ml) was applied as positive control. **(C)** A549 cells were infected with D39 with or without catalase (Cat) and D39Δ*spxB* (MOI = 200) at 1 and 5 h, *ISG15*, *OASL-1*, *RNF185* mRNA levels were determined by real-time PCR. NC, negative control. All data were presented as means ± SD from three independent experiments. ^∗^*P* < 0.05; ^∗∗^*P* < 0.01; ^∗∗∗^*P* < 0.001 was considered statistically significant and highly statistically significant differences, respectively; ns, not significant.

IFN-I production has been shown to be triggered by mtDNA through STING signaling (Fang et al., 2016). We examined the protein level of STING in A549 cells following different stimulations, including D39 with or without catalase, D39Δ*spxB* and H_2_O_2_. The results showed that both D39 infection and H_2_O_2_ stimulation up-regulated the expression of STING in A549 cells (Figure 5A), and the induction of STING was reduced by 20% by catalase treatment, a 40% reduction was observed after infection with D39Δ*spxB*. To further confirm that STING is responsible for inducing IFN-I expression in response to detecting mtDNA oxidized by S. *pn*-secreted H_2_O_2_, WT MEFs and STING knockout MEFs (MEFs sting−/−) were stimulated with D39, D39Δ*spxB*, and H_2_O_2_. Real-time PCR results demonstrated upregulation of *Ifnβ* and *Ifna4* in WT MEF cells, but not in MEF sting−/− cells (Figure 5B). Curiously, there was no change in the transcriptional level of the IFN-responsive gene, C-X-C motif chemokine 10 (*Cxcl10*), in either WT MEFs or MEF sting−/− cells upon stimulation with D39, D39Δ*spxB* or H_2_O_2_. These results demonstrated that STING is indispensable in *S. pn* H_2_O_2_-induced production of IFN-I in MEF cells.

Additionally, we also determined the expression of IFN-I-stimulated genes *ISG15* and *OASl-1*, as well as *RNF185* (which has been reported to positively regulate the STING signaling pathway) in A549 cells infected with D39 (Figure 5C) (Wang Q. et al., 2017). We found that *ISG15*, *OASl-1*, and *RNF185* were induced in A549 cells infected with D39 at 1 h, but not 5 h post-infection. Furthermore, the addition of catalase or infection with D39Δ*spxB* diminished the expression of these genes at 1 h. Taken together, these findings indicate that STING signaling is probably involved in inducing the expression of IFN-I in response to mtDNA damaged by *S. pn*-secreted H_2_O_2_.

## Discussion

In this study, we ascertained that *S. pn*-secreted H_2_O_2_ promoted IFNβ production in lung cells, which was mediated by mtDNA leakage from mitochondria damaged by H_2_O_2_. Neutralizing the H_2_O_2_ produced by *S. pn* H_2_O_2_ with catalase markedly attenuated mitochondrial malfunction and IFNβ expression, suggesting that targeting H_2_O_2_ during *S. pn* infection may offer therapeutic strategies.

There are several virulence factors of *S. pn* that are involved in the disease process. Specifically, *S. pn* is able to secrete substantial amounts of H_2_O_2_—up to a concentration of approximately 2 mM under aerobic conditions (Duane et al., 1993; Echlin et al., 2016; Lisher et al., 2017). Other *Streptococcus* species, such as *Streptococcus sanguis* (Sumioka et al., 2017), *Oral streptococci* (Matsushima et al., 2017), which have been reported to secrete H_2_O_2_. Several studies indicate that H_2_O_2_ secretion by most *Streptococcus* species is universal and indispensable. The production of H_2_O_2_ is dependent on the pyruvate oxidase gene *spxB*, which confers a selective advantage in co-colonization (Pesakhov et al., 2007; Regev-Yochay et al., 2007). Deletion of *spxB* results in a significant reduction in H_2_O_2_ to approximately 20% of level produced by WT *S. pn* (Echlin et al., 2016). However, *spxB* may also play other roles in the virulence of *S. pn* as there are several different serotypes. It has been shown that the virulence of a *spxB* knockout mutant of strain D39 (serotype 2) is attenuated in a murine model of nasopharyngeal colonization (Spellerberg et al., 1996), while a *spxB* mutant of *S. pn* serotype 1 is hypervirulent (Syk et al., 2014).

Research have reported that H_2_O_2_ inhibited cell migration in a dose-dependent manner, and this would impair airway epithelial cell repair (Hamada et al., 2016). And A549 cells exposed to H_2_O_2_ caused powerful LDH release and a necrotic phenotype rather than programmed cell death (Schmeck et al., 2004). These suggested that H_2_O_2_ is able to damage lung tissue. Some studies have supported that the toxic H_2_O_2_ secreted by *S. pn* could cause cellular oxidative stress and participate in cellular immune responses through different signaling pathways. A previous study reported that two pneumococcal toxins, Ply and H_2_O_2,_ led to mitochondrial damage and consequently caused apoptosis of brain cells (Braun et al., 2002). Recent studies have shown that *S. pn*-secreted H_2_O_2_ induced DNA damage and apoptosis in lung cells, and contributed to the genotoxicity and virulence of *S. pn* (Rai et al., 2015). Moreover, there are some reports showing that pneumococcal H_2_O_2_-induced stress signaling regulated the expression of inflammatory genes (Loose et al., 2015). However, others have reported that the pneumococci-induced oxidative stress was independent of *S. pn*- secreted H_2_O_2_ and Ply but depended on the pneumococcal autolysin LytA (Zahlten et al., 2015). Here, we showed that *S. pn*- secreted H_2_O_2_ alone was able to induce mitochondrial oxidative damage, impairing mtDNA replication and decreasing mtDNA content in lung cells. Moreover, H_2_O_2_ is a type of reactive oxygen species (ROS), and as such, is an important signaling molecule that mediates oxidative stress and cellular damage (Wible and Bratton, 2018). Previous studies on intestinal health have revealed that H_2_O_2_ upregulated intracellular and mitochondrial ROS expression (Jiang et al., 2017). However, whether H_2_O_2_ and the production of ROS induces mtDNA damage still needs to be further clarified.

The unique aspect of mitochondria is that it is the only source of DNA in cells that does not reside in the nucleus. mtDNA-mediated signaling is the basis of the host immune defense in several diseases. In atherosclerosis, oxidative damage and replication errors are the sources of mtDNA defects, which lead to mitochondrial dysfunction and directly promote atherosclerosis (Yu and Bennett, 2014). In intestinal ischemia reperfusion (I/R), mtDNA contributed to the early phase of I/R injury and amplified the inflammatory response (Yue et al., 2015; Hu et al., 2018). During the process of metabolic stress–induced endothelial inflammation and insulin resistance, palmitic acid caused mtDNA leakage into the cytoplasm, and activated STING signaling to mediate the intercellular adhesion molecule (ICAM)-1 expression and endothelial inflammation (Mao et al., 2017). Interestingly, mtDNA could activate several innate immune pathways including TLR9, NLRP3 and STING signaling pathways in the mammalian immune responses (Fang et al., 2016).

IFN-I are pleiotropic cytokines produced in response to viruses, bacteria, and parasites. In bacterial infection, lipopolysaccharide (LPS) or bacterial nucleic acids are recognized by innate immune receptors, triggering IFN-I production (Boxx and Cheng, 2016). IFN-I induce differential effects on the immune response of the host. *Listeria monocytogenes* was reported to induce IFNβ expression, suppress the production of IFNγ and TNFα, thereby promoting infection (Auerbuch et al., 2004; Rayamajhi et al., 2010). In contrast, *S. pn* DNA initiated an IFN-I cascade that contributed to pneumococcal clearance, and this process played an important part in the host defense against pneumococci by inhibiting bacterial transmigration (Parker et al., 2011; LeMessurier et al., 2013). Interestingly, our data showed that exposure of alveolar epithelial cells to H_2_O_2_ produced by *S. pn* was sufficient to induce mtDNA leakage into the cytoplasm and induce IFNβ production.

Growing evidence has indicated that STING signaling can be triggered by DNA from pathogens or damaged self-DNA in the cytoplasm (Barber, 2015). Hartlova et al. (2015) demonstrated that unrepaired DNA lesions promoted the production of IFN-I via the STING signaling pathway, which strengthened anti-microbial immunity. In acute pancreatitis, STING sensed self-DNA from dying acinar cells and promoted inflammation (Zhao et al., 2018). Our results confirmed that *S. pn*- secreted H_2_O_2_ resulted in both the production of IFN-I and the activation of IFN-I-stimulated genes, *ISG15* and *OASl-1*, and *RNF185* in A549 cells. We verified that deletion of STING notably impaired the expression IFN-I in MEF cells. These data suggested that STING signaling may play an indispensable role in the production of IFN-I induced by *S. pn* H_2_O_2_.

A previous study showed that IFNβ induced ROS production in human myotubes, which contributed to mitochondrial dysfunction and resulted in muscle impairment and continued inflammation in dermatomyositis (Meyer et al., 2017). Likewise, another study reported that caspases controlled antiviral immunity through cGAS cleavage during inflammasome activation, resulting in reduced IFN-I expression, revealing a negative feedback that regulates the output of DNA-sensing pathways (Wang Y. et al., 2017). However, further research is needed in order to elucidate the end result of IFN-I production by *S. pn*-secreted H_2_O_2_. Specifically, studies need to determine if this signaling pathway ultimately favors bacterial clearance or aggravates host cell apoptosis.

Overall, our findings demonstrated that *S. pn*-secreted H_2_O_2_ induced mtDNA leakage into the cytoplasm, which resulted in the activation of the IFN-I, and this process may be mediated via STING signaling. We also confirmed that *S. pn* H_2_O_2_ was sufficient to mediate mitochondrial oxidative stress, which underscores the importance of mitochondrial homeostasis during the host immune defense. In summary, we have identified a novel signaling mechanism that may serve as a potential target for controlling *S. pn* infection.

## Data Availability

All datasets generated for this study are included in the manuscript and/or the supplementary files.

## Author Contributions

YG, WX, and XZ conceived and designed the experiments. YG, XD, SY, and HW (fourth author) performed the experiments. YG, XZ, and HL analyzed the data. YG, XH, and HW (corresponding author) wrote the manuscript. WX and HW (corresponding author) reviewed and edited the manuscript.

## Conflict of Interest Statement

The authors declare that the research was conducted in the absence of any commercial or financial relationships that could be construed as a potential conflict of interest.
